# Supplementary material for: A Likelihood Approach for Real-Time Calibration of Stochastic Compartmental Epidemic Models
Source: PLoS Comput Biol. 2017 Jan 17;13(1):e1005257. doi: 10.1371/journal.pcbi.1005257 (PMC5240920; doi:10.1371/journal.pcbi.1005257)
Supplement: S1 File — (TAR.GZ) [file pcbi.1005257.s014.tar.gz › HSPH_Online-SI-Revision/MSS10/n10-severe/n10-severe_NEW2_table8.pdf]

1	newly 3 weeks spec	53.	3.93	{0., 12.}	92.5849	1.76601	newly 3 weeks cumul	226.	63.09	{14., 107.}	72.0841	0.674288	newly 5 weeks spec	40.	0.23	{0., 0.}	99.425	2.49394	newly 5 weeks cumul	307.	64.35	{14., 109.}	79.0391	0.801304	newly 43 weeks cumul	452.	64.35	{14., 109.}	85.7633	0.969304
2	newly 3 weeks spec	48.	50.79	{41., 61.}	14.5208	0.0598854	newly 3 weeks cumul	239.	200.55	{178., 226.}	16.1464	0.0779364	newly 5 weeks spec	25.	31.02	{21., 41.}	31.04	0.114039	newly 5 weeks cumul	302.	271.09	{238., 300.}	11.1755	0.0526387	newly 45 weeks cumul	375.	376.71	{321., 448.}	11.2453	0.0485105
3	newly 3 weeks spec	7.	10.13	{2., 18.}	73.8571	0.281488	newly 3 weeks cumul	64.	52.98	{31., 73.}	24.6563	0.136669	newly 5 weeks spec	1.	3.62	{0., 10.}	324.	0.723931	newly 5 weeks cumul	67.	62.96	{35., 92.}	27.8806	0.139991	newly 47 weeks cumul	68.	68.58	{36., 105.}	32.1471	0.149787
4	newly 3 weeks spec	37.	27.07	{18., 38.}	29.973	0.166542	newly 3 weeks cumul	140.	122.93	{96., 149.}	15.9071	0.0767809	newly 5 weeks spec	17.	12.89	{5., 21.}	35.2353	0.212449	newly 5 weeks cumul	186.	154.58	{122., 194.}	19.4086	0.0969026	newly 42 weeks cumul	207.	181.1	{133., 238.}	18.4831	0.0905854
5	newly 3 weeks spec	63.	81.85	{2., 163.}	110.778	0.635201	newly 3 weeks cumul	261.	325.82	{81., 575.}	84.3218	0.363082	newly 5 weeks spec	22.	54.05	{0., 119.}	235.409	1.1552	newly 5 weeks cumul	329.	445.82	{82., 823.}	100.796	0.423626	newly 42 weeks cumul	383.	688.31	{82., 1344.}	149.856	0.525198
6	newly 3 weeks spec	37.	14.64	{4., 24.}	60.4865	0.526128	newly 3 weeks cumul	120.	76.12	{44., 109.}	38.2833	0.238344	newly 5 weeks spec	13.	5.31	{0., 11.}	65.4615	0.747327	newly 5 weeks cumul	158.	90.43	{51., 136.}	43.3481	0.283553	newly 43 weeks cumul	173.	98.51	{57., 152.}	44.0636	0.290572
7	newly 3 weeks spec	17.	40.78	{25., 56.}	140.353	0.362106	newly 3 weeks cumul	136.	171.91	{138., 209.}	27.8456	0.102255	newly 5 weeks spec	12.	21.36	{9., 34.}	85.1667	0.250779	newly 5 weeks cumul	163.	223.09	{170., 279.}	38.1043	0.134159	newly 42 weeks cumul	196.	279.29	{203., 366.}	43.8827	0.149579
8	newly 3 weeks spec	10.	9.49	{3., 16.}	44.5	0.289866	newly 3 weeks cumul	74.	49.51	{27., 72.}	34.7973	0.228499	newly 5 weeks spec	3.	3.6	{0., 10.}	112.	0.720756	newly 5 weeks cumul	80.	59.3	{28., 91.}	31.425	0.20488	newly 46 weeks cumul	83.	64.31	{29., 100.}	33.5542	0.211861
9	newly 3 weeks spec	28.	14.19	{5., 25.}	51.3214	0.370152	newly 3 weeks cumul	137.	75.74	{46., 108.}	44.7299	0.278916	newly 5 weeks spec	9.	5.05	{0., 10.}	61.2222	0.601888	newly 5 weeks cumul	177.	88.92	{50., 130.}	49.8079	0.324379	newly 50 weeks cumul	178.	94.83	{51., 144.}	47.1517	0.304556
10	newly 3 weeks spec	9.	5.65	{0., 10.}	50.1111	0.490211	newly 3 weeks cumul	56.	32.26	{15., 52.}	43.9286	0.304003	newly 5 weeks spec	0.1	1.7	{0., 4.}	1694.	0.744854	newly 5 weeks cumul	64.	37.23	{16., 60.}	44.2344	0.308742	newly 50 weeks cumul	67.	39.21	{16., 61.}	45.6567	0.318864
11	newly 3 weeks spec	0.1	1.49	{0., 5.}	1536.	0.428363	newly 3 weeks cumul	7.	15.1	{3., 32.}	143.714	0.357963	newly 5 weeks spec	0.1	0.09	{0., 0.}	184.	0.0420412	newly 5 weeks cumul	7.	15.69	{3., 34.}	152.143	0.364194	newly 49 weeks cumul	7.	15.84	{3., 34.}	154.286	0.365178
12	newly 3 weeks spec	18.	16.09	{6., 28.}	38.9444	0.215381	newly 3 weeks cumul	97.	84.32	{57., 115.}	21.732	0.110364	newly 5 weeks spec	11.	5.86	{0., 13.}	58.	0.580709	newly 5 weeks cumul	121.	99.62	{62., 136.}	25.157	0.133391	newly 49 weeks cumul	154.	107.49	{62., 159.}	33.4481	0.193113
13	newly 3 weeks spec	27.	31.53	{21., 43.}	27.7407	0.107733	newly 3 weeks cumul	156.	153.91	{130., 177.}	10.1987	0.0451896	newly 5 weeks spec	15.	13.36	{5., 23.}	36.2667	0.1973	newly 5 weeks cumul	190.	187.26	{157., 218.}	10.7684	0.0479564	newly 46 weeks cumul	207.	209.91	{169., 256.}	12.6908	0.0550614
14	newly 3 weeks spec	5.	8.6	{1., 19.}	108.4	0.390713	newly 3 weeks cumul	40.	45.64	{18., 72.}	44.	0.200217	newly 5 weeks spec	0.1	2.94	{0., 8.}	2920.	0.945218	newly 5 weeks cumul	41.	52.72	{21., 85.}	53.9024	0.218724	newly 48 weeks cumul	41.	57.03	{21., 92.}	63.1951	0.237745
15	newly 3 weeks spec	2.	6.48	{2., 12.}	237.	0.519077	newly 3 weeks cumul	22.	38.56	{22., 55.}	82.	0.257997	newly 5 weeks spec	2.	1.91	{0., 5.}	101.5	0.730071	newly 5 weeks cumul	26.	44.06	{23., 61.}	78.0769	0.252027	newly 52 weeks cumul	28.	46.46	{24., 67.}	75.7857	0.246897
16	newly 3 weeks spec	8.	2.55	{0., 5.}	70.625	0.804198	newly 3 weeks cumul	58.	10.81	{2., 18.}	81.3621	0.832068	newly 5 weeks spec	4.	1.32	{0., 4.}	74.5	0.965648	newly 5 weeks cumul	73.	13.93	{2., 26.}	80.9178	0.841939	newly 48 weeks cumul	90.	16.77	{2., 33.}	81.3667	0.88193
17	newly 3 weeks spec	89.	101.49	{85., 123.}	16.3708	0.0635312	newly 3 weeks cumul	412.	442.21	{407., 480.}	8.60922	0.0353697	newly 5 weeks spec	62.	53.4	{36., 69.}	18.7097	0.0953223	newly 5 weeks cumul	564.	568.99	{507., 633.}	6.68262	0.0288261	newly 48 weeks cumul	769.	690.59	{595., 781.}	11.6996	0.0553063
18	newly 3 weeks spec	67.	38.75	{27., 50.}	42.1642	0.250729	newly 3 weeks cumul	293.	170.12	{146., 197.}	41.9386	0.239113	newly 5 weeks spec	37.	18.11	{11., 26.}	51.0541	0.33899	newly 5 weeks cumul	379.	215.56	{179., 246.}	43.124	0.248833	newly 46 weeks cumul	562.	260.79	{204., 305.}	53.5961	0.338625
19	newly 3 weeks spec	35.	110.38	{95., 123.}	215.371	0.496417	newly 3 weeks cumul	159.	435.14	{408., 463.}	173.673	0.436793	newly 5 weeks spec	32.	65.48	{53., 80.}	104.625	0.30634	newly 5 weeks cumul	221.	585.57	{552., 619.}	164.964	0.422734	newly 42 weeks cumul	292.	794.85	{734., 854.}	172.209	0.434063
20	newly 3 weeks spec	92.	18.55	{2., 42.}	79.837	0.943452	newly 3 weeks cumul	401.	184.48	{93., 279.}	53.995	0.372476	newly 5 weeks spec	59.	1.97	{0., 6.}	96.661	2.22993	newly 5 weeks cumul	544.	193.01	{103., 287.}	64.5202	0.486922	newly 42 weeks cumul	745.	194.27	{103., 288.}	73.9235	0.621436
21	newly 3 weeks spec	36.	13.53	{4., 24.}	62.5833	0.515069	newly 3 weeks cumul	108.	70.27	{46., 96.}	36.2315	0.210252	newly 5 weeks spec	14.	4.81	{0., 12.}	69.7857	0.776208	newly 5 weeks cumul	144.	83.08	{50., 118.}	43.0972	0.26458	newly 50 weeks cumul	181.	90.78	{52., 136.}	49.9779	0.328426
22	newly 3 weeks spec	13.	21.26	{8., 34.}	80.	0.253616	newly 3 weeks cumul	94.	104.42	{64., 147.}	29.7447	0.120911	newly 5 weeks spec	3.	7.35	{0., 15.}	180.333	0.518293	newly 5 weeks cumul	123.	124.09	{76., 182.}	26.9512	0.120413	newly 48 weeks cumul	123.	140.94	{81., 210.}	35.5935	0.139964
23	newly 3 weeks spec	73.	12.24	{0., 24.}	83.2329	1.04971	newly 3 weeks cumul	250.	102.42	{44., 157.}	59.032	0.431877	newly 5 weeks spec	30.	1.96	{0., 6.}	93.4667	1.81413	newly 5 weeks cumul	319.	109.9	{47., 169.}	65.5486	0.508893	newly 47 weeks cumul	375.	110.96	{47., 170.}	70.4107	0.575822
24	newly 3 weeks spec	15.	7.98	{2., 15.}	52.8	0.404022	newly 3 weeks cumul	84.	49.69	{29., 70.}	41.2262	0.254447	newly 5 weeks spec	11.	3.21	{0., 7.}	72.4545	0.892937	newly 5 weeks cumul	110.	57.94	{33., 83.}	47.5818	0.308437	newly 48 weeks cumul	115.	62.7	{33., 91.}	46.487	0.301941
25	newly 3 weeks spec	0.1	2.12	{0., 7.}	2136.	0.653379	newly 3 weeks cumul	2.	16.51	{3., 34.}	729.5	0.798002	newly 5 weeks spec	0.1	0.39	{0., 1.}	468.	0.152076	newly 5 weeks cumul	2.	18.03	{3., 40.}	805.5	0.818578	newly 51 weeks cumul	2.	18.62	{3., 40.}	835.	0.82329
26	newly 3 weeks spec	24.	15.32	{6., 25.}	40.8333	0.273747	newly 3 weeks cumul	102.	79.06	{53., 108.}	26.5098	0.145219	newly 5 weeks spec	5.	5.78	{0., 13.}	82.	0.505348	newly 5 weeks cumul	124.	93.54	{55., 125.}	28.9032	0.161891	newly 50 weeks cumul	128.	101.79	{55., 144.}	28.1953	0.156651
27	newly 3 weeks spec	35.	40.58	{23., 59.}	29.7714	0.117078	newly 3 weeks cumul	192.	191.59	{158., 221.}	11.0052	0.0487094	newly 5 weeks spec	19.	17.85	{8., 28.}	32.3684	0.167419	newly 5 weeks cumul	236.	237.02	{184., 281.}	12.4661	0.0549669	newly 51 weeks cumul	284.	272.54	{206., 339.}	14.4014	0.0662881
28	newly 3 weeks spec	7.	6.34	{0., 14.}	68.5714	0.580777	newly 3 weeks cumul	31.	36.89	{9., 63.}	56.4839	0.256095	newly 5 weeks spec	4.	1.77	{0., 7.}	96.25	1.17494	newly 5 weeks cumul	37.	42.69	{9., 77.}	56.4595	0.266364	newly 50 weeks cumul	43.	46.	{9., 82.}	54.0465	0.273332
29	newly 3 weeks spec	0.1	2.84	{0., 7.}	2814.	0.975934	newly 3 weeks cumul	12.	20.08	{5., 37.}	93.	0.300587	newly 5 weeks spec	0.1	0.65	{0., 2.}	718.	0.239078	newly 5 weeks cumul	12.	22.43	{5., 42.}	111.25	0.327585	newly 52 weeks cumul	12.	23.65	{5., 45.}	121.417	0.335762
30	newly 3 weeks spec	0.1	3.43	{0., 9.}	3380.	1.12652	newly 3 weeks cumul	4.	22.06	{6., 37.}	454.5	0.66754	newly 5 weeks spec	0.1	0.92	{0., 3.}	964.	0.390526	newly 5 weeks cumul	4.	24.94	{6., 45.}	526.5	0.707133	newly 52 weeks cumul	4.	25.85	{6., 46.}	549.25	0.716727
31	newly 3 weeks spec	16.	14.56	{0., 30.}	61.875	0.500381	newly 3 weeks cumul	78.	85.15	{42., 129.}	36.0641	0.176574	newly 5 weeks spec	25.	5.74	{0., 15.}	78.72	1.2597	newly 5 weeks cumul	122.	99.63	{42., 155.}	33.0902	0.211084	newly 49 weeks cumul	141.	110.07	{42., 177.}	37.0426	0.242121
32	newly 3 weeks spec	66.	16.68	{2., 31.}	74.9091	0.818029	newly 3 weeks cumul	192.	106.39	{51., 167.}	44.849	0.302747	newly 5 weeks spec	25.	5.33	{0., 14.}	78.68	1.26019	newly 5 weeks cumul	260.	121.56	{56., 207.}	53.2462	0.380875	newly 42 weeks cumul	338.	128.72	{56., 217.}	61.9172	0.474496
33	newly 3 weeks spec	53.	13.84	{3., 26.}	73.8868	0.720695	newly 3 weeks cumul	200.	119.23	{60., 175.}	40.935	0.25883	newly 5 weeks spec	33.	2.9	{0., 10.}	91.2121	1.78542	newly 5 weeks cumul	277.	128.07	{61., 191.}	53.7653	0.369392	newly 42 weeks cumul	389.	129.6	{61., 198.}	66.6838	0.512906
34	newly 3 weeks spec	62.	5.65	{0., 14.}	90.8871	1.74602	newly 3 weeks cumul	247.	76.59	{14., 145.}	68.9919	0.634547	newly 5 weeks spec	32.	0.5	{0., 0.}	98.4375	2.37404	newly 5 weeks cumul	321.	78.81	{14., 151.}	75.4486	0.739981	newly 42 weeks cumul	401.	78.93	{14., 151.}	80.3167	0.836066
35	newly 3 weeks spec	19.	14.3	{6., 23.}	36.9474	0.23481	newly 3 weeks cumul	121.	71.78	{43., 99.}	40.6777	0.252998	newly 5 weeks spec	16.	5.36	{0., 13.}	68.5	0.792294	newly 5 weeks cumul	158.	86.13	{53., 123.}	45.4873	0.292806	newly 49 weeks cumul	183.	94.24	{57., 136.}	48.6885	0.322865
36	newly 3 weeks spec	68.	53.06	{38., 66.}	24.1471	0.126748	newly 3 weeks cumul	286.	222.61	{189., 250.}	22.1643	0.111277	newly 5 weeks spec	63.	28.57	{18., 39.}	54.6508	0.361201	newly 5 weeks cumul	404.	290.52	{246., 337.}	28.0891	0.146101	newly 45 weeks cumul	496.	361.31	{305., 427.}	27.1593	0.141754
37	newly 3 weeks spec	40.	22.32	{10., 34.}	44.7	0.295037	newly 3 weeks cumul	178.	103.12	{67., 136.}	42.0674	0.252635	newly 5 weeks spec	13.	10.52	{2., 20.}	48.6154	0.308434	newly 5 weeks cumul	219.	128.29	{82., 176.}	41.4201	0.249783	newly 48 weeks cumul	249.	149.2	{90., 213.}	40.6667	0.246724
38	newly 3 weeks spec	60.	4.54	{0., 13.}	92.4333	1.72052	newly 3 weeks cumul	237.	81.6	{29., 135.}	65.5696	0.533085	newly 5 weeks spec	33.	0.34	{0., 0.}	98.9697	2.40836	newly 5 weeks cumul	316.	83.02	{29., 137.}	73.7278	0.651484	newly 48 weeks cumul	375.	83.06	{29., 137.}	77.8507	0.72568
39	newly 3 weeks spec	66.	9.5	{0., 23.}	85.6061	1.32322	newly 3 weeks cumul	292.	119.69	{34., 197.}	59.0445	0.464913	newly 5 weeks spec	58.	1.59	{0., 7.}	97.2586	2.33423	newly 5 weeks cumul	396.	125.24	{34., 213.}	68.3737	0.580854	newly 44 weeks cumul	641.	125.71	{34., 213.}	80.3885	0.788689
40	newly 3 weeks spec	57.	11.06	{1., 21.}	80.5965	0.977953	newly 3 weeks cumul	264.	90.52	{43., 137.}	65.7121	0.521085	newly 5 weeks spec	47.	2.49	{0., 8.}	94.7021	1.99962	newly 5 weeks cumul	347.	97.62	{43., 149.}	71.8674	0.611619	newly 44 weeks cumul	489.	99.23	{43., 154.}	79.7076	0.754349
41	newly 3 weeks spec	16.	8.04	{1., 15.}	58.	0.532879	newly 3 weeks cumul	51.	49.02	{28., 72.}	29.0196	0.136079	newly 5 weeks spec	0.1	2.35	{0., 7.}	2344.	0.791426	newly 5 weeks cumul	55.	55.52	{30., 85.}	31.4545	0.140958	newly 52 weeks cumul	59.	59.06	{30., 94.}	34.8814	0.156619
42	newly 3 weeks spec	25.	7.8	{2., 15.}	68.88	0.631963	newly 3 weeks cumul	108.	45.56	{27., 70.}	57.8148	0.407647	newly 5 weeks spec	2.	2.56	{0., 6.}	115.	0.680971	newly 5 weeks cumul	125.	52.62	{27., 81.}	57.904	0.413221	newly 43 weeks cumul	125.	56.03	{28., 86.}	55.448	0.391354
43	newly 3 weeks spec	9.	7.07	{1., 15.}	54.5556	0.387403	newly 3 weeks cumul	69.	39.14	{18., 61.}	44.4348	0.299175	newly 5 weeks spec	6.	2.27	{0., 6.}	73.5	0.981089	newly 5 weeks cumul	83.	45.52	{20., 73.}	46.4337	0.31721	newly 51 weeks cumul	83.	47.65	{20., 77.}	45.747	0.309086
44	newly 3 weeks spec	52.	17.89	{4., 33.}	65.75	0.608069	newly 3 weeks cumul	260.	113.45	{57., 170.}	56.3654	0.392615	newly 5 weeks spec	27.	5.92	{0., 15.}	78.0741	1.16332	newly 5 weeks cumul	320.	129.51	{66., 196.}	59.5281	0.426465	newly 45 weeks cumul	421.	140.11	{68., 206.}	66.7197	0.513436
45	newly 3 weeks spec	23.	48.97	{35., 62.}	113.348	0.318154	newly 3 weeks cumul	107.	246.68	{209., 275.}	130.542	0.360592	newly 5 weeks spec	11.	19.19	{11., 28.}	79.5455	0.239274	newly 5 weeks cumul	130.	296.79	{249., 331.}	128.3	0.35603	newly 48 weeks cumul	143.	331.33	{277., 373.}	131.699	0.362009
46	newly 3 weeks spec	21.	22.19	{7., 40.}	52.619	0.288373	newly 3 weeks cumul	150.	122.65	{56., 185.}	30.9	0.178878	newly 5 weeks spec	19.	7.78	{0., 19.}	63.6842	0.793002	newly 5 weeks cumul	189.	143.25	{59., 215.}	33.2646	0.204684	newly 47 weeks cumul	195.	152.51	{59., 242.}	32.8564	0.200404
47	newly 3 weeks spec	36.	30.12	{19., 42.}	27.1111	0.14049	newly 3 weeks cumul	193.	139.26	{103., 174.}	28.3523	0.152821	newly 5 weeks spec	17.	14.9	{5., 25.}	39.1765	0.224052	newly 5 weeks cumul	233.	175.38	{126., 219.}	26.0687	0.139436	newly 48 weeks cumul	246.	207.88	{138., 272.}	22.3984	0.115319
48	newly 3 weeks spec	58.	122.17	{104., 140.}	110.638	0.320413	newly 3 weeks cumul	260.	504.45	{474., 531.}	94.0192	0.287379	newly 5 weeks spec	33.	66.53	{53., 78.}	101.606	0.299102	newly 5 weeks cumul	358.	662.62	{618., 704.}	85.0894	0.266812	newly 45 weeks cumul	432.	845.92	{774., 914.}	95.8148	0.290924
49	newly 3 weeks spec	33.	26.62	{16., 38.}	26.303	0.14459	newly 3 weeks cumul	141.	123.89	{91., 159.}	18.461	0.0922206	newly 5 weeks spec	29.	13.33	{4., 25.}	55.4138	0.45313	newly 5 weeks cumul	202.	155.4	{110., 205.}	24.7723	0.133771	newly 47 weeks cumul	230.	181.53	{116., 256.}	26.3609	0.142552
50	newly 3 weeks spec	0.1	17.09	{10., 26.}	16990.	2.19835	newly 3 weeks cumul	33.	95.24	{72., 114.}	188.606	0.454089	newly 5 weeks spec	0.1	6.16	{1., 12.}	6074.	1.61403	newly 5 weeks cumul	33.	111.27	{82., 137.}	237.182	0.520027	newly 52 weeks cumul	33.	121.46	{89., 158.}	268.061	0.554792
